# Neurophysiological Findings in Neuronal Ceroid Lipofuscinoses

**DOI:** 10.3389/fneur.2022.845877

**Published:** 2022-02-25

**Authors:** Marina Trivisano, Alessandro Ferretti, Costanza Calabrese, Nicola Pietrafusa, Ludovica Piscitello, Giusy Carfi' Pavia, Federico Vigevano, Nicola Specchio

**Affiliations:** Rare and Complex Epilepsy Unit, Department of Neuroscience, Bambino Gesù Children's Hospital, IRCCS, Full Member of European Reference Network EpiCARE, Rome, Italy

**Keywords:** neuronal ceroid lipofuscinoses, EEG, photoparoxysmal response, intermittent photic stimulation, visual evoked potentials, electroretinogram, neurophysiological findings

## Abstract

Neuronal ceroid lipofuscinoses (NCLs) are a heterogeneous group of neurodegenerative diseases, characterized by progressive cerebral atrophy due to lysosomal storage disorder. Common clinical features include epileptic seizures, progressive cognitive and motor decline, and visual failure, which occur over different time courses according to subtypes. During the latest years, many advances have been done in the field of targeted treatments, and in the next future, gene therapies and enzyme replacement treatments may be available for several NCL variants. Considering that there is rapid disease progression in NCLs, an early diagnosis is crucial, and neurophysiological features might have a key role for this purpose. Across the different subtypes of NCLs, electroencephalogram (EEG) is characterized by a progressive deterioration of cerebral activity with slowing of background activity and disappearance of spindles during sleep. Some types of heterogeneous abnormalities, diffuse or focal, prevalent over temporal and occipital regions, are described in many NCL variants. Photoparoxysmal response to low-frequency intermittent photic stimulation (IPS) is a typical EEG finding, mostly described in CLN2, CLN5, and CLN6 diseases. Visual evoked potentials (VEPs) allow to monitor the visual functions, and the lack of response at electroretinogram (ERG) reflects retinal neurodegeneration. Taken together, EEG, VEPs, and ERG may represent essential tools toward an early diagnosis of NCLs.

## Introduction

Neuronal ceroid lipofuscinoses (NCLs) are a heterogeneous group of autosomal recessive neurodegenerative disorders, characterized by progressive cerebral atrophy due to widespread accumulation of autofluorescent storage material within lysosomes (1–5). NCLs are caused by defective lysosomal processing enzymes or receptors (3–8). So far, 14 variants of NCLs are reported; they share some common clinical features including epileptic seizures, progressive cognitive and motor decline, and visual failure; all those symptoms occur over different time courses according to subtypes (9, 10).

Neuronal ceroid lipofuscinoses represent the most common cause of dementia in children (5, 8, 11), and the incidence varies worldwide from 1:12,500 to 1:100,000 (9).

Neuronal ceroid lipofuscinoses can be often misdiagnosed at the onset because of the appearance of non-specific presenting symptoms; therefore, the diagnosis may be delayed (5).

Clinical management is mostly palliative, although, in the recent years, many efforts have been done to identify targeted treatments, i.e., enzymatic replacement therapy (ERT) and genetic therapy for each of NCL variants (7, 12–16). Nevertheless, a targeted treatment with human recombinant enzyme is currently available only for CLN2 disease (12).

Considering the rapid disease progression (17) and the development of novel targeted treatments, early diagnosis remains crucial. Early diagnosis is also useful for genetic counseling to avoid family odyssey toward different hospitals looking for diagnosis and to avoid repetition of useless investigations (18). Next-generation sequencing techniques are successful in the identification of genetic childhood epilepsies and early detection of CLN2 disease (19). A recent study supported the value of target re-sequencing in patients with genetic childhood epilepsies, suggesting that this technique may be successful in the early detection of patients with CLN2 (19).

In this context, neurophysiological data may have a key role in the diagnosis of NCLs. Electroencephalogram (EEG) and evoked potentials may be of particular importance for this purpose, since they can provide critical information at a relatively early phase of the disease and are easy to carry out in a short time and without excessive costs.

This review aims to provide neurophysiological findings in NCLs, highlighting the neurophysiologic typical features of NCLs as well as what may allow differential diagnosis with developmental and epileptic encephalopathies (DEEs) due to other etiologies.

Two authors (MT and AF) performed a search in PubMed and EMBASE databases, from 1970 to 2021, using the following keywords: “ceroid lipofuscinosis” and “photoparoxysmal response,” “electroencephalogram”, “evoked potentials,” and “electroretinogram.” They looked for experimental and clinical studies and reviews. No meta-analyses were found.

## Neurophysiological Studies

### Electroencephalogram

Epilepsy is one of the cardinal symptoms of NCLs (5). The age at epilepsy onset may differ according to the NCLs variant, starting from the first months of life to adulthood; both focal and generalized seizures can be seen at onset (5). Epilepsy may represent the presenting symptom, as it happens in CLN2 and CLN6 diseases, or appear after visual loss or cognitive and motor decline, as in CLN3 and CLN5 diseases (20).

In the classic form of CLN1 disease, symptoms begin during infancy, and seizure onset is relatively early, typically between the age of 14 and 36 months. Additional phenotypes have been observed with late infantile, juvenile, and adult onset. In the latter cases, epilepsy typically begins several years after the initial symptom of visual impairment, following developmental regression and behavioral changes (20). Few data are available on EEG features in CLN1 disease (21, 22). It has been reported that a slowing of background activity and loss of sleep spindles is associated with high-voltage slow waves and spike and waves abnormalities (22). Between 5 and 12 years of age, a progressive flattening of cerebral activity consistent with the marked cortical atrophy due to massive neuronal death is evident (21). In summary, in CLN1 disease, changes of background activity have been distinguished in three stages: (1) decreasing of reactivity of the posterior rhythm to eye opening and closing; (2) decreasing of sleep spindles and subsequent disappearance; and (3) slowing and/or attenuation of EEG to inactivity, the so-called *vanishing* EEG pattern (22, 23).

In CLN2 disease, a late-infantile neuronal ceroid lipofuscinosis (LINCL), seizures often appear in an explosive fashion (24). Seizures are resistant to common anti-seizure medications (ASMs), and sodium channel blockers, such as carbamazepine, can even worsen seizure frequency and may increase myoclonus and ataxia (24).

**Figure 1:**
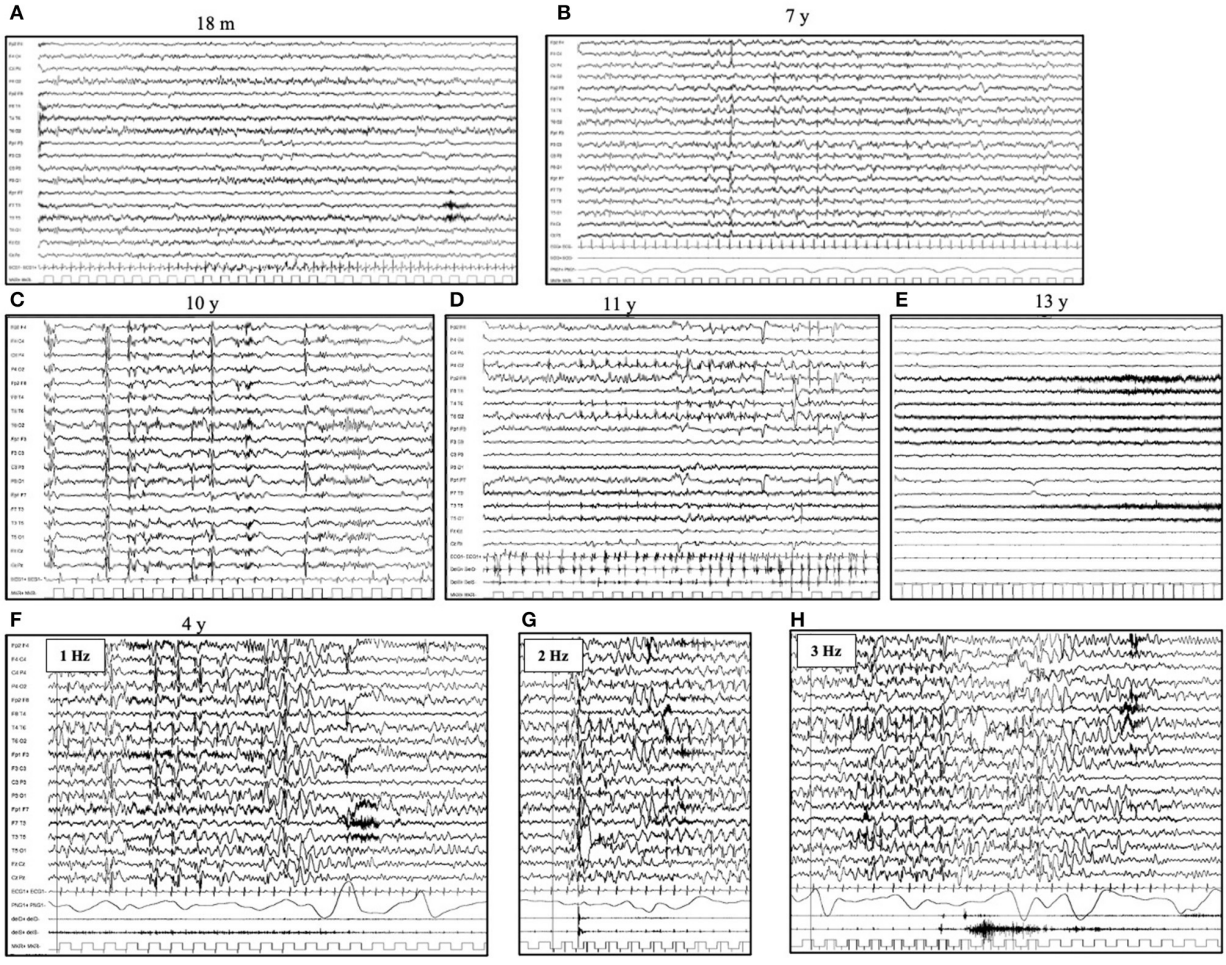
Electroencephalogram (EEG) evolution in CLN2 disease. Awake EEG **(A)** at the age of 18 months (pre-symptomatic phase) shows a normal background activity, with 8 Hz rhythm over bilateral posterior regions. No epileptiform abnormalities are evident. Awake EEG **(B)** at the age of 7 years shows a slow background activity and the lack of regional differentiation. There are diffuse and focal (mainly occipital) spikes and spike and waves abnormalities. During sleep **(C)**, the EEG (age of 10 years) shows the lack of sleep spindles and bursts of rapid spikes and poly-spikes, alternated with the suppression of cerebral activity. **(D)** Myoclonic status can occur in the advanced phases of diseases (age of 11 years) and **(E)** A diffuse flattening of cerebral activity with an extremely slow and low-voltage cerebral activity is typical of the latter stage. **(F–H)** Photoparoxysmal response in a 4-year-old girl affected by CLN2 disease. The flash-per-flash response is evident at the lower frequency of intermittent photic stimulation, at 1, 2, and 3 Hz (respectively **F–H**).

The first EEG is usually performed within the fourth year of life and shows a normal background activity, associated with focal or diffuse abnormalities in 75% of the patients. High-voltage focal slow waves are prevalent over the temporal and occipital regions (25–28). Photoparoxysmal response (PPR) to intermittent photic stimulation (IPS) delivered at a low frequency of stimulation is typically seen in patients with CLN2 during the first stage of the disease (27). With disease progression, the background activity becomes slow and without reactivity to eye opening (29, 30). No spindles are present during sleep recordings. Generalized, focal and multifocal epileptiform discharges characterized by irregular spikes and poly-spikes and waves become common; however, a posterior predominance persists (24) (Figures 1A–E).

In CLN3 disease, the juvenile neuronal ceroid lipofuscinosis (Spielmeyer–Vogt disease or Batten disease), the presenting manifestation is a progressive visual loss starting around the age of 6–8 years. Epilepsy usually starts at the age of 10 years. Seizures at onset are usually bilateral tonic–clonic, whereas focal seizures increase during adolescence, and they are mostly characterized by clonic manifestations. Myoclonic seizures are rarely reported in CLN3 disease. EEG, in the early stages of the disease, shows focal epileptiform abnormalities (before 10 years of age), whereas bilateral and multifocal epileptiform discharges are significantly more prevalent in the later stages, along with a progressive slowing of the background activity (31). PPR has not been reported as a prominent feature in CLN3 disease (31).

The CLN6 disease may start both during infancy and adulthood. In both cases, epilepsy is one of the presenting symptoms (32). Background activity is poorly organized during awake and sleep since the onset, associated with irregular slow spike and waves discharges (at about 2.5 Hz). PPR to lower frequency is an early neurophysiological finding. During the advanced stage of disease, the cerebral activity became extremely slow, and low-voltage and spike and waves discharge became rare, often replaced by single spikes with multifocal distribution (32).

In adult patients affected by CLN6, the background activity is almost preserved in the early stage of the disease. Epileptiform abnormalities are the most prevalent over the posterior regions. Myoclonus is typically induced by active movements or provoked by IPS at a low frequency of stimulus. Abnormal PPR persists until the advanced stages of the disease (32).

The CLN5 disease, also known as the Finnish variant, starts between the age of 2 and 6 years with clumsiness and mental decline (33, 34). Seizure occurs relatively late (median age of 8 years), and epilepsy has its major expression with myoclonic seizures between the age of 7 and 11 years. In addition, for this LINCL, it has been reported that a progressive slowing of background activity is associated with multifocal epileptiform discharges (spikes, multiple spikes, and spike and wave complexes) (34) and the presence of posterior spikes triggered by low-frequency IPS (33, 34).

**Figure 2:**
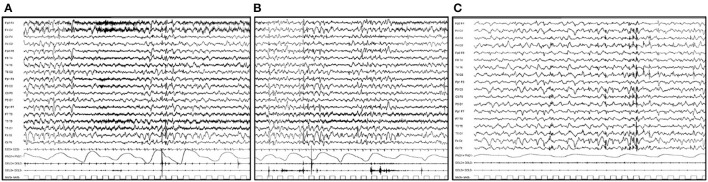
EEG features in a 6-year-old boy affected by CLN7 disease. **(A)** EEG trace shows a generalized slowing of background activity associated with diffuse or multifocal abnormalities. **(B)** Single myoclonic jerks involving the upper limbs with the EEG correlate of a diffuse single spike are evident. **(C)** During sleep, the cerebral activity is markedly slow and is associated with multifocal spike and spike and waves abnormalities, prevalent over bilateral posterior regions.

Regarding CLN7 disease, in few published patients, it has been reported that a progressive generalized slowing of background activity is associated with diffuse or multifocal abnormalities with occipital prevalence (Figures 2A–C). PPR has not been reported (35, 36).

In patients with CLN8, it has been described as a progressively slowing of background activity with focal and/or generalized abnormalities since the onset. PPR has rarely been reported (37).

Few cases of CLN11 have been reported (38, 39). EEG is characterized by a quite preserved background activity associated with rare paroxysms of diffuse spikes and slow waves, prevalent over bilateral parietal regions. PPR has not been detected in the three cases reported so far (38, 39).

Limited data have been reported on EEG features for the other NCL variants, from CLN9 to CLN14, which are the more recently identified forms. More cases need to be reported to understand their typical neurophysiological findings.

### Photoparoxysmal Response

Photoparoxysmal response in NCLs was firstly described in 1970 when it was reported in about 45% of patients (29). Later, other studies confirmed the presence of this peculiar neurophysiological finding (27, 32, 40, 41).

The susceptibility to PPR varies accordingly to the different diseases which are more prominent in the LINCLs, such as CLN2, CLN5, and CLN6 (Figures 1F–H). In CLN2 disease, PPR has been reported from 27 to 93% of patients (25, 27, 40, 41). This wide range may be explained by the lack of a universal approach of low frequency IPS in routine pediatric EEG recordings (24). In fact, in NCLs, as well as in other progressive myoclonic epilepsies, PPR is one of the earliest pieces of evidence of a neurodegenerative disorder, even before the onset of cognitive and motor regression (42). PPR to low-frequency stimulation has also been reported to occur in Lafora disease and mitochondrial diseases (43, 44).

In a recently reported series of 14 cases of CLN2, serial EEGs revealed a PPR in 93% of patients (27). PPR was evident since the first EEG, which was performed at 3.6 (3.1–4.0) years, in 43% of patients; it was documented at low (1–3 Hz) frequencies of stimulation in 69% of patients and has acquired the form of a flash-per-flash response in 69% of patients (27). Moreover, in the advanced stages of the disease, PPR was associated with massive myoclonic jerks (27). In about half of patients (54%), PPR disappears over time (25). PPR changes over time reflect the gray matter changes due to the progression of neurodegenerative disease (27).

The PPR seen in the NCLs is characterized by an occipital spike and waves response to the photic stimuli. However, it should also be highlighted that not in all NCL variants do patients have the same susceptibility to PPR; it has been reported more frequently in CLN2, CLN5, and CLN6 diseases (24, 27, 32, 33) and seems to not be a prominent feature in CLN1, CLN3, CLN8, and CLN11 diseases (21, 31, 37, 38). For the remaining NCLs, we do not have sufficient data about PPR to find out conclusive data.

On the other hand, the lack of the characteristic IPS response cannot rule out an NCL disease (45). The implementation of IPS, including IPS at low frequencies of stimulation as part of a standard EEG, may be useful as an early disease marker if associated with other clinical findings (24, 45, 46).

### Flash and Pattern-Reversal Visual Evoked Potentials

**Figure 3:**
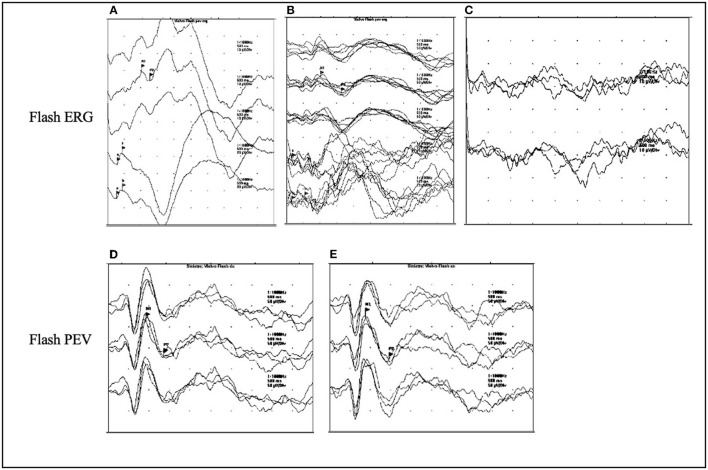
ERG and VEPs in CLN2 disease. **(A–C)** The ERG flash evolution in a patient affected with CLN2 at the ages of 3.5, 5.5, and 7 years. The response is normal at the onset of disease at the age of 3.5 years **(A)**, at the age of 5.5 years, there is a reduction of the amplitude of ERG **(B)**, which turns to be absent at the age of 7 years **(C)**. **(D,E)** The presence of giant flash VEPs in the left and right eyes in a 4-year-old patient.

Few data are available in the literature regarding visual evoked potentials (VEPs) changes during the disease course in NCLs. What is known is that with disease progression, the so-called *giant* VEPs appear (Figures 3D,E). They are abnormally broad and of high amplitude, and their presence is a marker of cortical hyperexcitability, similar to PPR (32).

Abnormal VEPs have been found in about 75% of patients with CLN2 at a median age of 4.5 years (25). Furthermore, a more recent study on patients with CLN2 evidenced that most patients (89%) had an early and high-amplitude pattern reversal in VEPs, whereas few patients showed a bifid waveform, which is associated with a central scotoma, indicative of maculopathy or macular pathway dysfunction such as optic atrophy (47).

With further disease progression, the amplitude of VEP decreases, as it happens in other neurodegenerative disorders (48, 49).

The pattern-reversal VEP waveform is preferred to the flash VEP waveform, which shows a wide inter-individual variability. On the contrary, the pattern-reversal VEP has a relatively constant single positive peak throughout life and is a strong index of macular pathway function (47).

Variability of results may be due to the retrospective nature of the studies and the different ages and phases of the disease in which visual tests have been performed (25, 41, 47).

### Electroretinogram

Vision-related problems are one of the cardinal signs of NCLs and, as in CLN3, are often an early sign, appearing prior to motor and mental deterioration (5). This is due to the accumulation of storage material into the retina, leading to its degeneration (50, 51).

Electroretinogram (ERG) represents the tool that allows to monitor the involvement of retina, and its use progressively disappears with disease progression (Figures 3A–C) at different ages according to the NCL variants (50). ERG is currently used to characterize the physiological changes in the degenerating retina in patients affected by NCLs. It allows to identify the retinopathy, which consists of symmetrical cone-rod dystrophy (47, 50).

In patients with CLN2, it has been demonstrated that changes in ERG appear even before the macular disruption on optical coherence tomography (OCT) from the age of 4 years and 10 months (47). Abnormal ERG has been reported in 66% of patients at a median age of 4.5 years (25). However, it is important to underlie that the flattening of ERG does not necessarily imply the total loss of retinal function, if some cortical VEP functions persist (29).

In CLN5 disease, retinal degeneration has been confirmed between the age of 6 and 10 years, when ERG has been found abolished in most of the patients (33, 34).

The use of ERG might be implemented in the future to evaluate the efficacy of experimental treatments with intravitreal therapies.

### Somatosensory Evoked Potentials

Somatosensory evoked potentials (SEPs) have been poorly investigated in NCL diseases. There are sporadic cases, across different forms of NCLs (21, 32, 33, 52), where it is highlighted that the presence of high-amplitude evoked potential, *giant* SEPs, which are the expressions of cortical hyperexcitability due to neuronal degeneration (32).

Giant SEPs are also a typical and specific marker of patients with cortical myoclonus, which is one of the main clinical features of NCLs (38).

## Differential Diagnosis

Neurophysiological investigations can also be useful to differentiate NCLs from other DEEs. In detail, LINCLs should be differentiated from those DEEs with epilepsy onset between 2 and 6 years of age, mainly characterized by myoclonic seizures. Among these, it is important to consider epilepsy with myoclonic-atonic seizures (EMA) and Lennox–Gastaut syndrome (LGS), which are characterized by epilepsy with multiple types of seizures, intellectual disability, and drug resistance (24).

The EEG is particularly useful for this purpose because, in NCLs, there is a slowing of background activity with progressive loss of differentiation and disappearance of spindles during sleep, whereas in EMA, although with the presence of many epileptiform abnormalities, the background activity is preserved (53). Moreover, a poly-graphic study might highlight that, in EMA, myoclonic events mainly involve proximal, axial muscles, whereas, in NCLs, myoclonus involves mainly distal, segmental muscles (38, 53).

The LGS is otherwise characterized by specific EEG abnormalities, which are diffuse spike-and-slow wave complexes at 2.5 Hz during awake, and poly-spikes during sleep (54). In both syndromes, PPR is rarely reported and, when described, it is not induced by low-frequency IPS (53, 54).

On the other hand, PPR is reported in some epileptic syndromes of infancy, and among these, The Dravet syndrome (DS) is most frequent (55). Different from the majority of NCLs, in DS, epilepsy starts within the first year of life. In addition, in DS, PPR is not typically induced by low-frequency IPS and does not have the no characteristics of the flash-per-flash PPR (55).

During the last decades, technological advances have driven genetic discovery in epilepsy and increased the understanding of the molecular mechanisms of many epileptic disorders, in some cases providing targets for precision medicine (19, 56). Nevertheless, phenotyping and neurophysiological characterization are still critical for the diagnosis and better management of neurological symptoms.

## Conclusions

**Figure 4:**
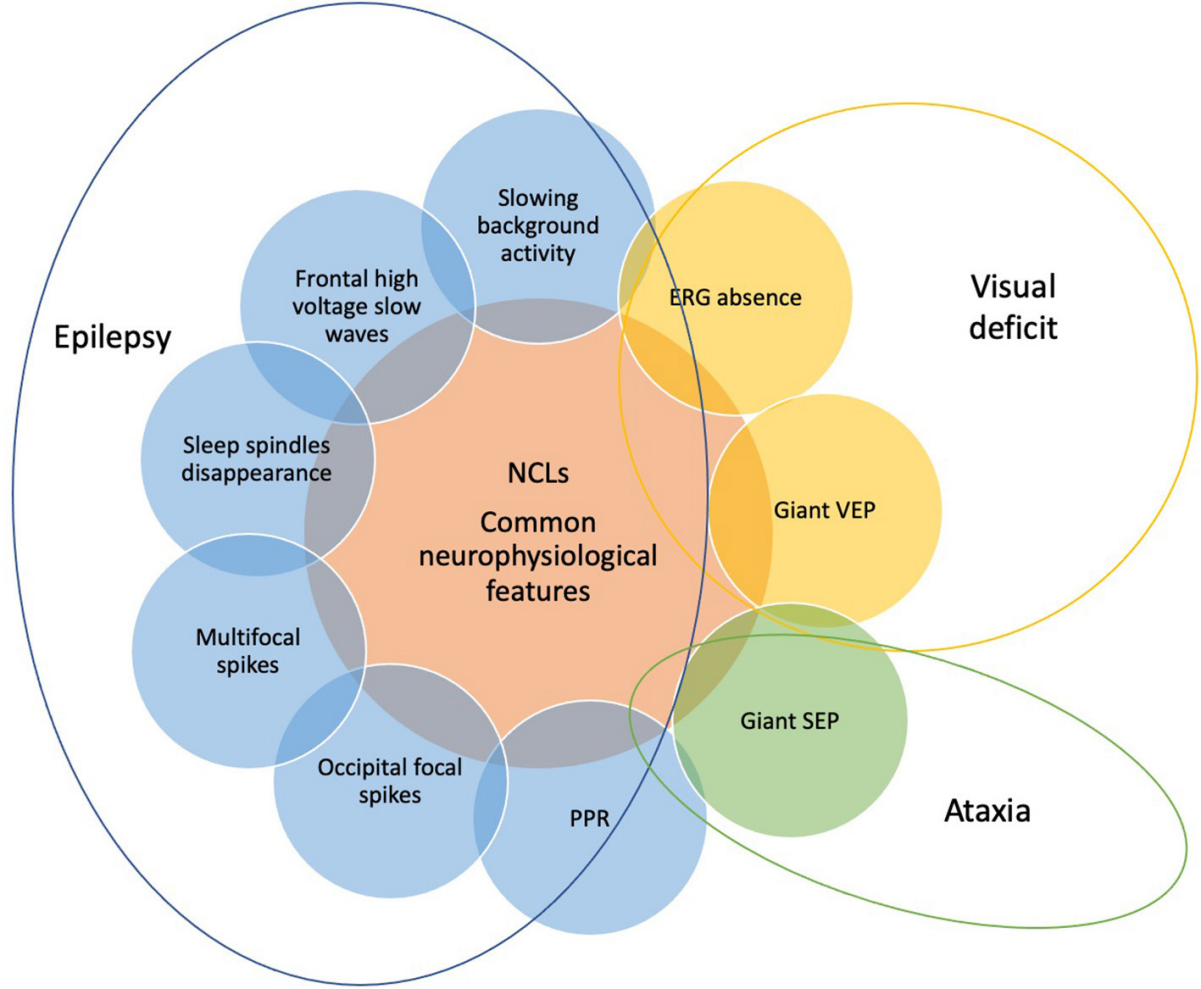
The common neurophysiological features of NCLs include EEG, visual evoked potentials (VEPs), somatosensory evoked potentials (SEPs), and electroretinogram findings. They can vary according to the different subtypes of NCLs and the age of patients. Each neurophysiological finding (slowing background activity, disappearance of sleep spindles, frontal high-voltage slow waves, occipital focal spikes, multifocal spikes, PPR, giant VEPs and SEP, and ERG absence) is the expression of one of the three main symptoms of NCLs, which are epilepsy, visual deficit, and ataxia and allows to monitor their progression.

An appropriate definition of neurophysiological features of NCLs is crucial for the possible role that they may have in the early diagnosis of such diseases. There are some features (Figure 4) that are common to different subtypes of NCLs, such as the progressive slowing of background activity, the disappearance of sleep spindles during sleep, and the presence of some type of heterogeneous abnormalities, such as bursts of diffuse or focal slow waves prevalent over temporal and occipital regions and diffuse spike and wave paroxysmal discharges (25–27).

Sensitivity to low-frequency IPS is a hallmark of neurodegenerative diseases such as NCLs and may be useful as an early marker if associated with other clinical findings (27). This peculiar EEG activation may be missed due to the lack of standardization of the test, which should be implemented with low-frequency stimulation, starting from 1 Hz (27).

The VEPs allow to monitor the visual pathway function, and the lack of visual response at ERG reflects retinal neurodegeneration.

Taken together, EEG, VEPs, and ERG may represent essential tools that can address the clinicians toward an early diagnosis of NCL disease. Although treatment remains essentially symptomatic in NCLs, together with palliative, supportive, and rehabilitative measures, in the near future, ERT and gene therapies may be available and earlier diagnosis will be mandatory. In this context, the use of NGS-based approaches results important for the early identification of patients with NCL, allowing a timely adoption of the most accurate treatment strategies.

## Author Contributions

MT and AF prepared the first draft of the manuscript. NS and FV coordinated the job and supervised critically the manuscript and reviewed the final version of the manuscript. CC did the reference search and provided support for the discussion. LP and GC prepared the figures and contributed to the figure legends and reviewed critically the manuscript. NP reviewed critically the manuscript and contributed to the final draft. All authors contributed to the article and approved the submitted version.

## Conflict of Interest

The authors declare that the research was conducted in the absence of any commercial or financial relationships that could be construed as a potential conflict of interest.

## Publisher's Note

All claims expressed in this article are solely those of the authors and do not necessarily represent those of their affiliated organizations, or those of the publisher, the editors and the reviewers. Any product that may be evaluated in this article, or claim that may be made by its manufacturer, is not guaranteed or endorsed by the publisher.
